# A Critical Role for IL-17RB Signaling in HTLV-1 Tax-Induced NF-κB Activation and T-Cell Transformation

**DOI:** 10.1371/journal.ppat.1004418

**Published:** 2014-10-23

**Authors:** Alfonso Lavorgna, Masao Matsuoka, Edward William Harhaj

**Affiliations:** 1 Department of Oncology, Sidney Kimmel Comprehensive Cancer Center, Johns Hopkins School of Medicine, Baltimore, Maryland, United States of America; 2 Laboratory of Virus Control, Institute for Virus Research, Kyoto University, Kyoto, Japan; University of Pennsylvania School of Medicine, United States of America

## Abstract

Human T-cell leukemia virus type 1 (HTLV-1) infection is linked to the development of adult T-cell leukemia (ATL) and the neuroinflammatory disease HTLV-1 associated myelopathy/tropical spastic paraparesis (HAM/TSP). The HTLV-1 Tax protein functions as a potent viral oncogene that constitutively activates the NF-κB transcription factor to transform T cells; however, the underlying mechanisms remain obscure. Here, using next-generation RNA sequencing we identified the IL-25 receptor subunit IL-17RB as an aberrantly overexpressed gene in HTLV-1 immortalized T cells. Tax induced the expression of IL-17RB in an IκB kinase (IKK) and NF-κB-dependent manner. Remarkably, Tax activation of the canonical NF-κB pathway in T cells was critically dependent on IL-17RB expression. IL-17RB and IL-25 were required for HTLV-1-induced immortalization of primary T cells, and the constitutive NF-κB activation and survival of HTLV-1 transformed T cells. IL-9 was identified as an important downstream target gene of the IL-17RB pathway that drives the proliferation of HTLV-1 transformed cells. Furthermore, IL-17RB was overexpressed in leukemic cells from a subset of ATL patients and also regulated NF-κB activation in some, but not all, Tax-negative ATL cell lines. Together, our results support a model whereby Tax instigates an IL-17RB-NF-κB feed-forward autocrine loop that is obligatory for HTLV-1 leukemogenesis.

## Author Summary

The retrovirus HTLV-1 is the causative agent of an aggressive lymphoproliferative disorder known as adult T-cell leukemia (ATL). The HTLV-1 Tax regulatory protein constitutively activates the host NF-κB transcription factor to promote T-cell proliferation, survival and cell transformation. However, it remains unknown precisely how Tax persistently activates NF-κB in T cells. In this study, we used next-generation sequencing to identify genes that were differentially expressed upon HTLV-1 infection and immortalization of primary T cells. We found that IL-17RB, the receptor for the IL-25 cytokine, was highly induced in HTLV-1 transformed T cells and was required for NF-κB activation, cell proliferation and survival. Tax induced the expression of IL-17RB and established a positive feedback loop together with IL-25 that triggered persistent NF-κB activation and the upregulation of IL-9 and other genes critical for T-cell proliferation and survival. IL-17RB was also overexpressed in a subset of acute ATL patient specimens and therefore may potentially be targeted by monoclonal antibodies as a novel ATL therapy.

## Introduction

The retrovirus human T-cell leukemia virus type 1 (HTLV-1) infects between 10–20 million people worldwide [1]. HTLV-1 is the etiological agent of the neuroinflammatory disease HTLV-1-associated myelopathy (HAM/TSP) and adult T-cell leukemia (ATL), a CD4+CD25+ T-cell malignancy [2], [3]. ATL develops in about 5% of HTLV-1-infected individuals after a long latent period spanning 40–60 years [4]. The HTLV-1 genome encodes the Tax protein that exerts pleiotropic roles and is an essential regulator of viral replication and oncogenic cell transformation [5]. Tax modulates the activation of several key signaling pathways and cell cycle proteins to enhance T-cell proliferation and survival. One of the key cellular targets important for transformation by Tax is the NF-κB transcription factor [6].

NF-κB is composed of heterodimeric DNA binding proteins consisting of RelA, c-Rel, RelB, p50 and p52 [7]. In the canonical NF-κB pathway, NF-κB heterodimers are sequestered in the cytoplasm by ankyrin-repeat containing inhibitory proteins including IκBα [8]. A wide variety of stimuli including stress signals, proinflammatory cytokines or virus infection activate the IKK kinase complex consisting of the catalytic subunits IKKα and IKKβ and the regulatory subunit IKKγ (also known as NEMO) [9]. IKKβ phosphorylates IκB proteins to trigger their ubiquitin-dependent degradation thus allowing NF-κB to enter the nucleus and activate target genes [10]. In the noncanonical NF-κB pathway, tumor necrosis factor receptor (TNFR) superfamily members including BAFF, lymphotoxin-β and CD40 promote proteasomal processing of the p100 (NF-κB2) precursor protein to yield p52, which forms transcriptionally active heterodimers with RelB. The NF-κB inducing kinase (NIK) is a key regulator of this pathway by activating IKKα homodimers which in turn phosphorylate p100 leading to its processing. Tax constitutively activates both the canonical and noncanonical NF-κB pathways, in part by interacting directly with NEMO and IKK [11]–[14]. There is evidence that Tax may require upstream signaling molecules such as the kinase TAK1 to activate canonical NF-κB signaling [15]. Although the proximal signaling components of TNFR and interleukin-1 receptor (IL-1R) are dispensable for Tax to activate NF-κB [16], whether Tax has usurped a distinct NF-κB pathway is unknown.

Tax activation of the canonical and noncanonical NF-κB pathways fosters the aberrant expression of anti-apoptotic and pro-proliferative genes that leads to oncogenesis. Tax mutants defective in NF-κB activation expressed in an infectious HTLV-1 molecular clone are impaired in the immortalization of primary T cells [17]. NF-κB is also required for the survival of HTLV-1 transformed cell lines and patient-derived ATL cells [18]. Therefore, HTLV-1 transformed cell lines and primary ATL leukemic specimens exhibit a strict “addiction” to NF-κB for survival and proliferation, thus establishing the NF-κB pathway as an attractive target for novel ATL therapeutics. However, since Tax expressing cells are vigorously targeted by cytotoxic T cells and other arms of the host immune response, the majority of ATL tumors exhibit downregulated or lost Tax expression by mutations within Tax or deletion or methylation of the 5′ viral long terminal repeat region (LTR) [19]. Thus, Tax likely plays more important roles in the early events of transformation via persistent NF-κB activation, inactivation of p53 and other tumor suppressors and induction of genomic instability and aneuploidy [5]. However, canonical and noncanonical NF-κB signaling remains constitutive in ATL despite the loss of Tax.

The interleukin 17 (IL-17) cytokine family consists of six members (IL-17A-F) that play essential roles in host immunity and inflammatory diseases. IL-17A is the signature IL-17 cytokine and binds to an IL-17RA/IL-17RC receptor complex to orchestrate the host response against bacterial and fungal infections [20]. IL-17A controls the expression of cytokines and chemokines that enhance neutrophil recruitment. Dysregulation of this pathway has been implicated in numerous autoimmune and metabolic diseases and cancer [21]. IL-17E (also known as IL-25) is essential for host defense against parasites by regulating expression of T helper 2 (Th2) cytokines including IL-4, IL-5 and IL-13 that promote eosinophil recruitment [22]. IL-25 has also been linked to allergic airway inflammation and asthma [23]. IL-25 is produced by diverse cell types such as epithelial cells, T cells, eosinophils, mast cells and basophils [24], [25]. IL-25 binds to a heterodimeric receptor composed of IL-17RA and IL-17RB, of which IL-17RB is the specific receptor subunit for IL-25 [26]. IL-17RB is highly expressed in kidney, liver and other peripheral organs as well as memory and effector T lymphocytes [27]. IL-17RB expression can be regulated by IL-4 and TGF-β, however the precise transcriptional regulatory control of IL-17RB is unknown. Upon binding to IL-25, IL-17RB recruits the Act1 (also known as CIKS) adaptor molecule via homotypic SEFIR (similar expression to fibroblast growth factor genes and IL-17R) domain interactions [28], [29]. Act1 activates the ubiquitin ligase TRAF6 and the kinase TAK1 that in turn triggers NF-κB and MAP kinase activation to induce type 2 cytokines IL-4, IL-5 and IL-13 as well as IL-9 [30], [31]. IL-17B also serves as a ligand for IL-17RB, albeit with a lower affinity for IL-17RB compared to IL-25 [32]. In addition to IL-17RB regulation of host defense and allergic airway disease, this pathway can be oncogenic if dysregulated. The *IL-17RB* locus is a common site of retroviral integration in murine myeloid leukemias, resulting in the upregulation of IL-17RB expression [33]. IL-17RB is also overexpressed in a subset of breast tumors and is associated with poor prognosis [34]. In breast cancer, IL-17RB engagement by IL-17B triggers TRAF6 recruitment to IL-17RB, NF-κB activation and induction of the *bcl-2* gene to inhibit apoptosis [34].

Although considerable progress has been made in our understanding of HTLV-1 oncogenesis, the precise mechanisms underlying HTLV-1-induced transformation remain unclear. Previous microarray studies have identified several anti-apoptotic, cell cycle and growth regulatory genes dysregulated by HTLV-1 [35]–[37]. However, due to experimental limitations of these studies and the advent of next-generation sequencing, RNA sequencing (RNA-Seq) has emerged as a powerful tool to evaluate gene expression, differential splicing, noncoding RNAs, RNA editing and gene fusions [38]. In this study, we used RNA-Seq to delineate the transcriptome of primary T lymphocytes immortalized by HTLV-1. This work led to the identification of IL-17RB as an aberrantly overexpressed gene in HTLV-1 transformed cells that was induced by the HTLV-1 Tax protein. Surprisingly, the IL-17RB pathway was required for constitutive NF-κB activation by Tax and in HTLV-1 transformed cell lines. Furthermore, IL-17RB was overexpressed in leukemic cells from acute ATL patients and was essential for NF-κB activation in a subset of Tax-negative ATL cell lines.

## Results

### Next-generation sequencing identifies the transcriptomes of primary T cells infected and immortalized by HTLV-1

**Figure 1:**
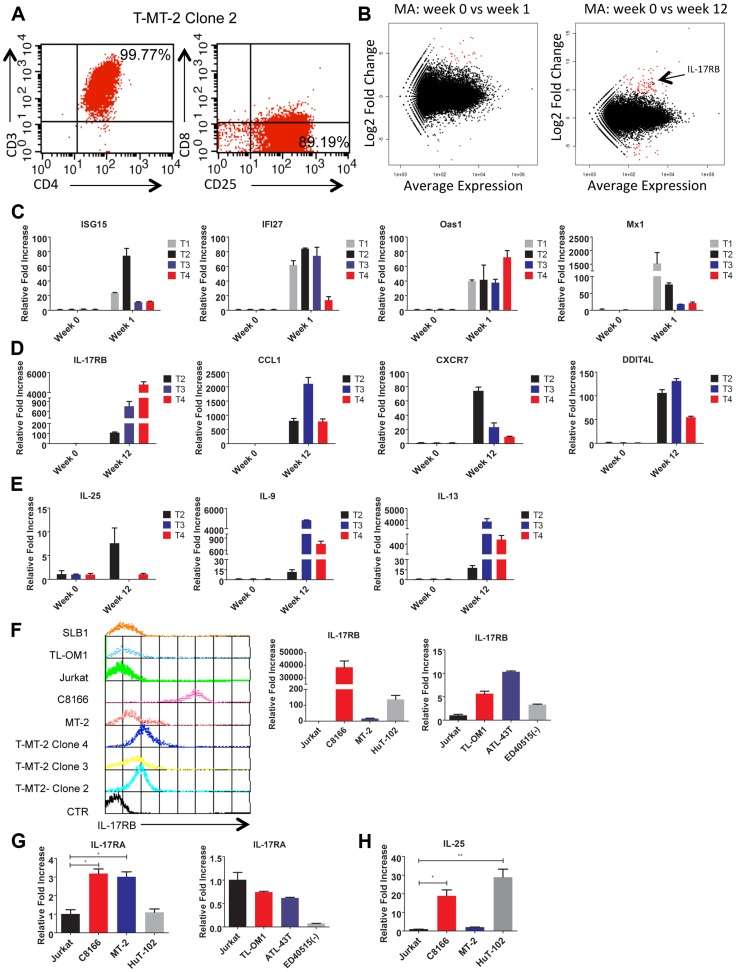
IL-17RB is overexpressed in HTLV-1 immortalized T cell clones and transformed cell lines. (**A**) Flow cytometric analysis of CD3/CD4/CD8/CD25 markers with T-MT-2 clone 2. (**B**) Differential gene expression of T cells at week 1 (top) and week 12 (bottom) compared to week 0 (parental T cells) analyzed using RNA-Seq and “DESeq” R package and plotted as an MA plot. DESeq plotMA displays differential expression (log-fold changes) versus expression strength (log average read count). (**C–E**) qRT-PCR of indicated mRNAs in T cell clones. (**F**) Flow cytometric analysis of IL-17RB was performed on the indicated immortalized HTLV-1 immortalized T-cell clones and HTLV-1+ cell lines (top). qRT-PCR of IL-17RB mRNA in HTLV-1+ and ATL cell lines (bottom). (**G, H**) qRT-PCR of IL-17RA and IL-25 mRNAs in HTLV-1+ and ATL cell lines.

To gain insight into the mechanisms of HTLV-1-induced T-cell immortalization, we used a well-established co-culture model [35], [39] whereby primary human CD4+ T cells were purified by immunomagnetic beads from normal donor peripheral blood mononuclear cells (PBMCs) and co-cultured with lethally irradiated HTLV-1 transformed MT-2 cells (to provide a source of HTLV-1). Primary T cells were consistently immortalized in the presence of MT-2 cells between 6–8 weeks. Control T cells cultured in the absence of MT-2 did not proliferate after 4 weeks and were no longer viable at that time. The co-culture assay was performed with T cells from 4 independent blood donors. Of the 4 co-cultures, all produced immortalized T cell clones, however clone #1 ceased proliferation unexpectedly and was excluded from further studies. The immortalized T cell clones (T-MT-2) #2-4 remained dependent on recombinant IL-2 for proliferation and expressed CD3, CD4 and CD25 cell surface markers (Figure 1A).

Total RNA was harvested from T-MT-2 clone #2 (week 12 after co-culture) for RNA-Seq analysis as well as parental primary T cells (week 0), and T cells after 1 week of co-culture. A pure population of viable cells was obtained from the co-culture after removal of dead cells using magnetic labeling and separation. MT-2 RNA was also included as a control for RNA-Seq to confirm that the immortalized T cells expressed a unique genetic signature and were not simply MT-2 contaminants. RNA-Seq and bioinformatics analysis were performed with a total number of reads of 65 million (week 0), 73 million (week 1), 44 million (week 12) and 52 million (MT-2). At 1 week after co-culture, the most abundant induced coding RNAs in the T cells were interferon-stimulated genes (ISGs) such as ISG15, IFI27, OAS1 and MX1 and these results were confirmed by real-time quantitative RT-PCR (qRT-PCR) (Figure 1C and Table S1). Conversely, the HTLV-1-immortalized T cells did not express ISGs, but rather expressed aberrant levels of genes regulating cell growth/cytokines (IL-17RB, IL-5, IL-9, IL-13, CADM1), DNA damage (DDIT4L), cell cycle (CDC14B, CCNA1), metabolism (glycerol kinase 2) and migration/chemokines (CCL1, CXCR7) (Table S2). Also, these immortalized T cells had a distinct genetic signature compared to MT-2 cells (Sequence read archive accession numbers SRS698576 and SRS698477). Notably, many of the aberrantly expressed genes, including IL-17RB, have not previously been linked to transformation by HTLV-1. IL-17RB was one of the most highly induced genes in HTLV-1 immortalized T cells (Figures 1B and S1 and Table S2). IL-17RB mRNA expression was sharply elevated in all 3 independent HTLV-1 immortalized T cell clones as shown by qRT-PCR (Figure 1D). IL-25, the high affinity ligand for IL-17RB, was expressed at variable levels in the three clones (Figure 1E). Aberrant expression of CCL1 (also known as I-309), CXCR7, DDIT4L, IL-9 and IL-13 in HTLV-1-immortalized clones was also confirmed by qRT-PCR (Figure 1D and E). The chemokine CCL1, shown previously to be overexpressed in ATL cells, functions in an anti-apoptotic autocrine loop [40]. Similarly, the chemokine receptor CXCR7 is induced by Tax and regulates the growth and survival of ATL cells [41]. Furthermore, Tax induction of both IL-9 and IL-13 may trigger the autocrine stimulation of HTLV-1 infected cells [42], [43]. Taken together, our RNA-Seq studies have confirmed the dysregulation of known targets of HTLV-1 transformation and have also identified genes, such as IL-17RB, not previously demonstrated to be induced by HTLV-1. Next, the cell surface expression of IL-17RB was examined in HTLV-1 transformed cell lines by flow cytometry. IL-17RB was highly expressed in the HTLV-1 immortalized T-cell clones and most HTLV-1-transformed cell lines, but not in Jurkat T cells (Figure 1F). IL-17RB mRNA was also overexpressed in varying degrees in HTLV-1 transformed and ATL cell lines (Figure 1F). Since IL-17RB forms heterodimers with IL-17RA, the expression of IL-17RA was examined in HTLV-1 transformed and ATL cell lines. IL-17RA and IL-25 mRNAs were also upregulated in a subset of HTLV-1 transformed and ATL cell lines (Figure 1G and H).

### IL-17RB expression is regulated by Tax and IKK

**Figure 2:**
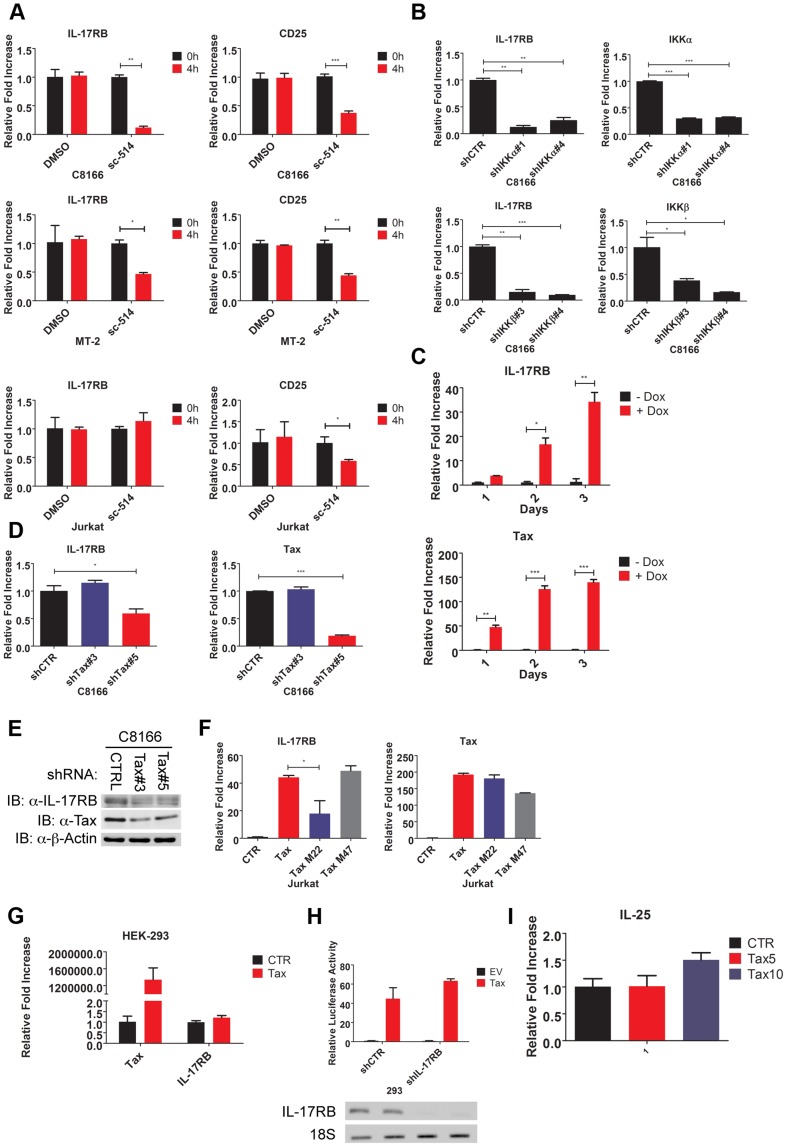
Essential roles for IKK and Tax in the induction of IL-17RB. (**A**) qRT-PCR of IL-17RB and CD25 mRNAs in C8166, MT-2 and Jurkat cells treated with the IKKβ inhibitor sc-514 (10 µM) for 4 h. (**B**) qRT-PCR of IL-17RB, IKKα and IKKβ mRNAs in C8166 cells transduced with lentiviruses expressing control, IKKα or IKKβ shRNAs. (**C**) qRT-PCR of IL-17RB and Tax mRNAs in Jurkat Tax Tet-On cells treated with Dox (1 µg/ml) for the indicated times. (**D**) qRT-PCR of IL-17RB and Tax mRNAs in C8166 cells transduced with lentiviruses expressing control or Tax shRNAs. (**E**) Western blot of IL-17RB, Tax and β-actin using lysates from C8166 cells transduced with lentiviruses expressing control or the indicated Tax shRNAs. (**F**) qRT-PCR of IL-17RB and Tax mRNAs in Jurkat cells transduced with lentiviruses expressing empty vector (CTR), Tax, Tax M22 or Tax M47. (**G**) qRT-PCR of IL-17RB or Tax mRNAs in 293 cells transduced with lentiviruses expressing empty vector (CTR) or Tax. (**H**) NF-κB luciferase assay with lysates from 293 cells transduced with lentiviruses expressing control (CTR) or IL-17RB shRNA, and then transfected with empty vector (EV) or pCMV4-Tax, together with an NF-κB luciferase reporter and pRL-TK. IL-17RB mRNA and 18S rRNA were detected by RT-PCR (bottom panel). (**I**) qRT-PCR of IL-25 mRNA in Jurkat cells transduced with lentiviruses expressing empty vector (CTR) or Tax at multiplicities of infection (MOIs) of 5 and 10.

A previous study reported a role for TGF-β and IL-4 in the upregulation of IL-17RB expression [31]. Since NF-κB is important for the proliferation and survival of HTLV-1 transformed cells, we hypothesized that NF-κB may regulate IL-17RB induction. Thus, the HTLV-1 transformed T-cell lines C8166 and MT-2 were treated with sc-514, a small molecule inhibitor of IKKβ, and qRT-PCR was performed for IL-17RB and the known NF-κB target gene CD25. Treatment with sc-514 significantly diminished the expression of IL-17RB and CD25 mRNAs in these cells (Figure 2A), thus supporting a role for IKKβ and NF-κB in the expression of IL-17RB in HTLV-1 transformed cells. However, sc-514 treatment had no effect on IL-17RB expression in Jurkat cells (Figure 2A). To provide further evidence for a role of IKK in the regulation of IL-17RB, recombinant lentiviruses expressing either control scrambled short hairpin RNA (shRNA) or two distinct shRNAs specific for IKKα or IKKβ were transduced into C8166 cells. Both IKKα and IKKβ shRNAs strongly suppressed their respective mRNAs as shown by qRT-PCR, and these shRNAs significantly inhibited IL-17RB expression (Figure 2B). Therefore, both IKKα and IKKβ regulate IL-17RB expression in C8166 cells.

The HTLV-1 Tax oncoprotein dysregulates the expression of specific cellular genes as part of its oncogenic mechanism [44]. To determine if Tax was involved in the induction of IL-17RB expression we used a Jurkat cell line inducible for Tax expression (Jurkat Tax Tet-On) by doxycycline (Dox) [45]. Jurkat Tax Tet-On cells were treated with Dox for 1, 2 and 3 days and mRNA was harvested for qRT-PCR for IL-17RB. Indeed, induction of Tax strongly upregulated IL-17RB mRNA (Figure 2C). Conversely, shRNA-mediated knockdown of Tax in C8166 cells diminished the expression of IL-17RB mRNA (Figure 2D). Knockdown of Tax also reduced the expression of IL-17RB protein in C8166 cells (Figure 2E). Thus, both gain-of-function and loss-of-function studies support the hypothesis that Tax is the HTLV-1-encoded protein that promotes the aberrant overexpression of IL-17RB.

Two commonly used Tax mutants M22 (Thr^130^Leu^131^->Ala^130^Ser^131^) and M47 (Leu^319^Leu^320^->Arg^319^Ser^320^) can be used to distinguish NF-κB or CREB-specific functions of Tax [46]. Tax M22 is defective for NF-κB and wild-type for CREB activation, whereas Tax M47 is defective for CREB and wild-type for NF-κB activation. Wild-type Tax, Tax M22 and Tax M47 were cloned into a lentiviral vector and recombinant Tax-expressing lentiviruses were used to transduce Jurkat T cells. Wild-type Tax and Tax M47 strongly upregulated IL-17RB mRNA expression as detected by qRT-PCR, however Tax M22 induction of IL-17RB was significantly diminished (Figure 2F). These data further support the notion that Tax requires NF-κB to induce IL-17RB expression. Interestingly Tax induction of IL-17RB was not observed in 293 cells suggesting that this event may be specific for T cells (Figure 2G). Tax activation of NF-κB was also independent of IL-17RB in 293 cells, since knockdown of IL-17RB in 293 cells had no effect on Tax activation of an NF-κB reporter (Figure 2H). Finally, the expression of IL-25 was not regulated by Tax in T cells, therefore Tax induces the expression of IL-17RB but not its high affinity ligand (Figure 2I).

### Tax requires IL-17RB to activate NF-κB in T cells

**Figure 3:**
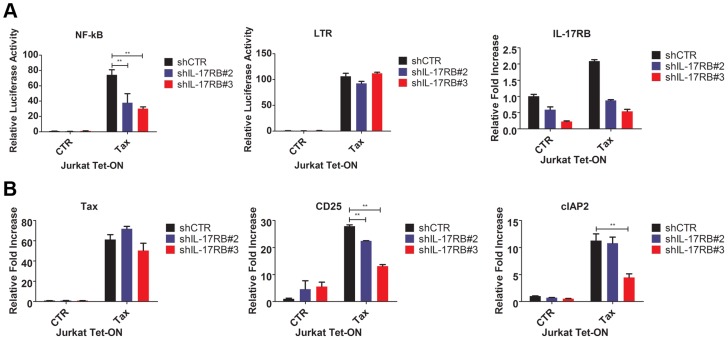
Tax requires IL-17RB for NF-κB activation in T cells. (**A**) NF-κB and HTLV-1 LTR luciferase assays and qRT-PCR of IL-17RB mRNA in Jurkat Tax Tet-On cells transduced with lentiviruses expressing control or IL-17RB shRNA. Cells were also transiently transfected with NF-κB and HTLV-1 LTR luciferase reporters and treated with Dox (1 µg/ml) for 24 h. (**B**) qRT-PCR of Tax, CD25 and cIAP2 mRNAs in Jurkat Tax Tet-On cells from panel (A).

Since IL-17RB signals to NF-κB, we next asked if Tax required IL-17RB to trigger NF-κB signaling in T cells. Jurkat Tax Tet-On cells were transduced with lentiviruses expressing control or IL-17RB shRNA, yielding ∼60–70% knockdown efficiency (Figure 3A). The cells were transiently transfected with NF-κB and HTLV-1 LTR reporters for dual-luciferase assays and also treated with Dox to activate Tax expression. Remarkably, Tax activation of NF-κB, but not the HTLV-1 LTR (which is CREB-mediated), was dependent on IL-17RB (Figure 3A). In agreement with these results, Tax induction of the NF-κB target genes, CD25 and cIAP2, was impaired when IL-17RB expression was suppressed with shRNAs (Figure 3B). Therefore, Tax induces IL-17RB expression to establish a positive feedback loop that is critical for Tax-induced NF-κB activation. Also, the requirement of IL-17RB for Tax-mediated NF-κB activation appears to be T-cell specific.

### IL-17RB and IL-25 are essential for HTLV-1-induced T-cell immortalization

**Figure 4:**
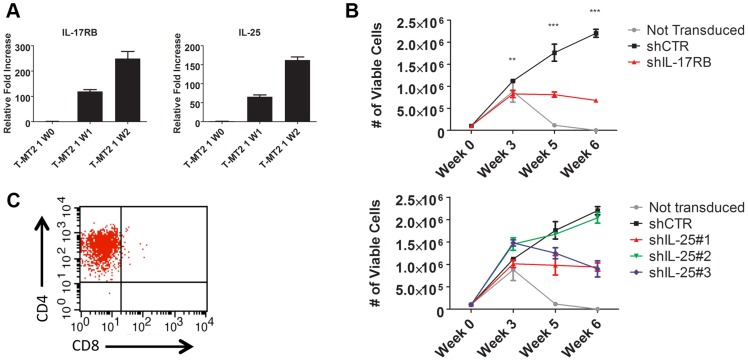
IL-17RB and IL-25 are essential for the HTLV-1-induced immortalization of T cells. (**A**) qRT-PCR of IL-17RB and IL-25 mRNAs in primary T cells co-cultured with lethally irradiated MT-2 cells after 0 (W0), 1 (W1) and 2 (W2) weeks. (**B**) *In vitro* immortalization assay using PBMCs transduced with lentiviruses expressing control (CTR), IL-17RB or IL-25 shRNAs and co-cultured with lethally irradiated MT-2 cells. (**C**) Flow cytometric analysis of CD4 and CD8 markers using immortalized PBMCs expressing control shRNA.

To determine the role of the IL-17RB pathway in the early events of HTLV-1 transformation of primary human T cells, we conducted an *in vitro* T-cell immortalization assay with irradiated MT-2 cells and PBMCs from normal donors as described earlier. In this co-culture model, expression of both IL-17RB and IL-25 were significantly increased in primary T cells at early times (1–2 weeks) after co-culture with MT-2 cells (Figure 4A). PBMCs were transduced with lentiviruses expressing control shRNA or shRNAs for IL-17RB or IL-25, co-cultured with irradiated MT-2 cells and puromycin was added to select for cells expressing shRNAs. Both IL-17RB and IL-25 were required for immortalization of primary T cells by HTLV-1 since cells expressing these shRNAs ceased to proliferate after 3 weeks of co-culture (Figure 4B). However, as expected PBMCs expressing control shRNA yielded immortalized CD4+ T cells after 8 weeks (Figure 4C). Taken together, these results suggest that both IL-25 and IL-17RB are required for the early events involved in the immortalization of primary T cells by HTLV-1.

### IL-17RB is essential for NF-κB signaling and the viability of HTLV-1 transformed T cell lines

**Figure 5:**
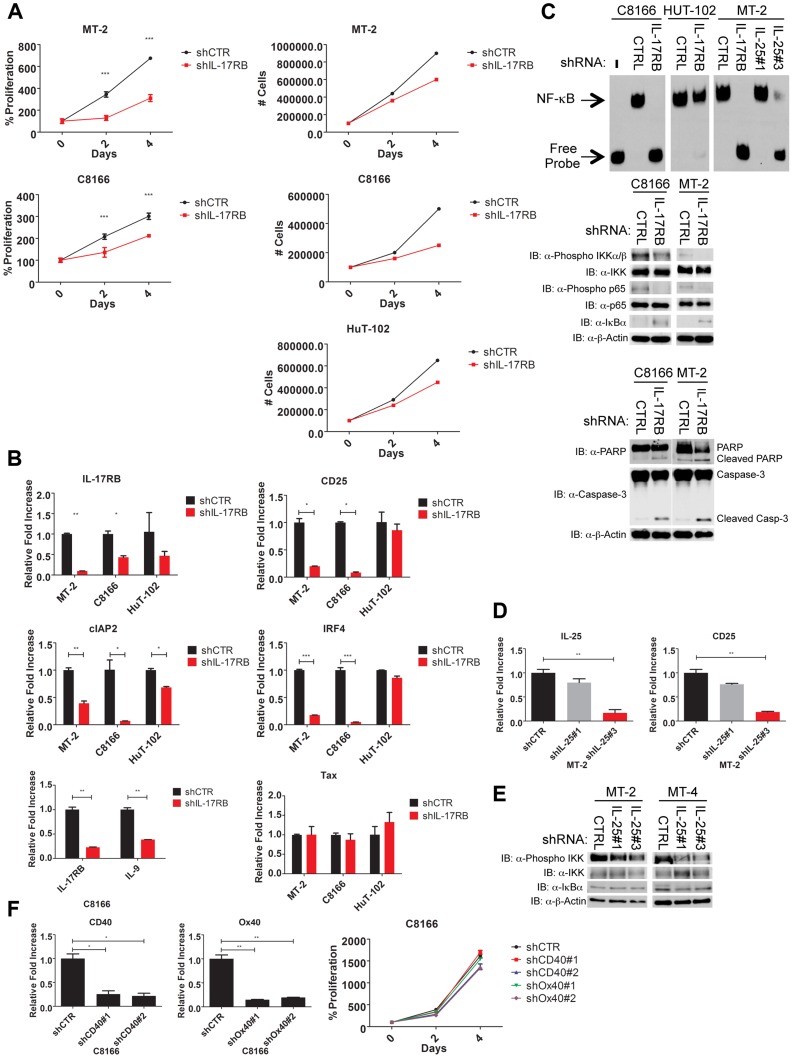
IL-17RB and IL-25 are essential for NF-κB activation and survival of HTLV-1 transformed cells. (**A**) Proliferation/viability assay of MT-2 and C8166 cells transduced with lentiviruses expressing control or IL-17RB shRNA using CellTiter-Glo (left). Cell counts of MT-2, C8166 and HUT-102 cells transduced with control or IL-17RB shRNA. (**B**) qRT-PCR of indicated mRNAs in HTLV-1 transformed cell lines transduced with lentiviruses expressing control or IL-17RB shRNA. (**C**) NF-κB EMSA using nuclear extracts from MT-2, C8166 and HUT-102 cells transduced with control, IL-17RB or IL-25 shRNAs (left). Western blots were performed with the indicated antibodies using whole-cell lysates from MT-2 and C8166 cells transduced with control, IL-17RB or IL-25 shRNAs (center and right). (**D**) qRT-PCR of CD25 and IL-25 mRNAs in MT-2 cells transduced with lentiviruses expressing control or IL-25 shRNA. (**E**) Western blots were performed with the indicated antibodies using whole cell lysates from MT-2 and MT-4 cells transduced with lentiviruses expressing control or IL-25 shRNAs. (**F**) qRT-PCR of CD40 and OX40 mRNAs in C8166 cells expressing CD40 or OX40 shRNAs (top). Proliferation/viability assay of C8166 cells expressing control, CD40 or OX40 shRNAs.

Because Tax required IL-17RB for efficient NF-κB activation and NF-κB is critical for the survival of T cells transformed by HTLV-1, we hypothesized that IL-17RB was essential for NF-κB activation and the viability of established HTLV-1 transformed cell lines. To address this notion, recombinant lentiviruses expressing control or IL-17RB shRNAs were transduced into three distinct Tax-expressing HTLV-1 transformed cell lines (C8166, MT-2 and HUT-102). A total of three independent shRNAs to IL-17RB or scrambled control shRNA were expressed in these cell lines and selected with puromycin. Efficient knockdown of IL-17RB was confirmed by qRT-PCR in MT-2 and C8166 cells (Figure 5B). The CellTiter-Glo Luminescent Cell Viability kit was used to quantify cellular ATP levels to determine cell viability and proliferation. Knockdown of IL-17RB significantly reduced the viability and proliferation of HTLV-1 transformed cell lines (Figure 5A). Next, we examined expression of the NF-κB target genes CD25, cIAP2, IRF4 and IL-9 by qRT-PCR. The expression of each of these genes was significantly attenuated upon IL-17RB knockdown in C8166 and MT-2 cells (Figure 5B). Importantly, Tax expression was unaffected by IL-17RB knockdown in these cell lines (Figure 5B).

An NF-κB DNA binding electrophoretic mobility shift assay (EMSA) was next performed with nuclear extracts from C8166, MT-2 and HUT-102 cells expressing control or IL-17RB shRNA. NF-κB DNA binding was completely abrogated upon IL-17RB suppression in C8166 and MT-2 cells, but not HUT-102 likely due to inefficient lentiviral transduction (Figure 5C). IL-25 was also suppressed by shRNAs in MT-2 cells and shRNA#3 was effective in reducing IL-25 expression (Figure 5D). Knockdown of IL-25 with this shRNA also significantly attenuated NF-κB DNA binding and the expression of CD25 (Figure 5C and D). NF-κB signaling can also be monitored with phospho-specific antibodies for IKK and p65 since these proteins are phosphorylated upon activation. Phosphorylation of IKK and p65 was constitutive in C8166, MT-2 and MT-4 cells but reduced upon knockdown of IL-17RB or IL-25 (Figure 5C and E). IκBα protein was increased upon suppression of IL-17RB (Figure 5C), likely reflecting enhanced stability due to a loss of IKK-induced phosphorylation and proteolysis. IL-17RB knockdown also triggered an apoptotic response in HTLV-1 transformed cells as revealed by PARP and caspase 3 cleavage (Figure 5C). The TNFR cell surface receptors CD40 and OX40 activate NF-κB, are strongly induced by Tax and are overexpressed in HTLV-1 transformed cell lines [47], [48]. However, knockdown of either CD40 or OX40 had no effect on the proliferation or viability of C8166 cells (Figure 5F). Thus, IL-17RB is a receptor that appears to be uniquely required for NF-κB signaling and the survival of HTLV-1 transformed cells.

### IL-9 is a downstream target gene of IL-17RB that controls the proliferation of HTLV-1 transformed T cells

**Figure 6:**
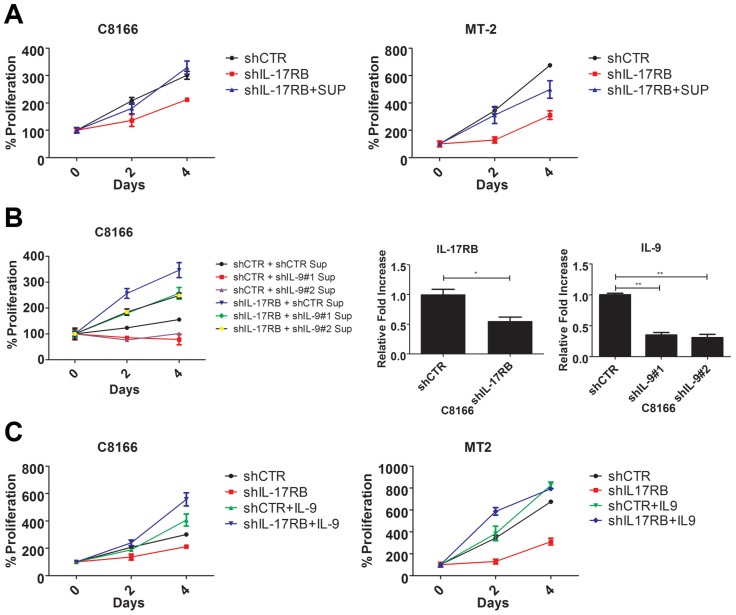
IL-9 is a key target gene downstream of IL-17RB that regulates the proliferation of HTLV-1 transformed cells. (**A**) Proliferation/viability assay of C8166 and MT-2 cells transduced with lentiviruses expressing control or IL-17RB shRNA using CellTiter-Glo. Cells knocked down for IL-17RB were grown in conditioned media (SUP) from the corresponding cell lines. (**B**) Proliferation/viability assay of C8166 cells transduced with lentiviruses expressing control or IL-17RB shRNA. Cells with suppressed IL-17RB expression were grown in conditioned media (Sup) from C8166 expressing control (CTR) or the indicated IL-9 shRNAs (top panel). qRT-PCR of IL-17RB and IL-9 mRNAs in C8166 cells transduced with lentiviruses expressing IL-17RB or IL-9 shRNAs (bottom panel). (**C**) Proliferation/viability assay of C8166 and MT-2 cells transduced with lentiviruses expressing control or IL-17RB shRNA, in the presence or absence of recombinant IL-9 (20 ng/ml) for the indicated times.

Our earlier results indicated that HTLV-1 immortalized T-cell clones expressed aberrant levels of IL-9 (Figure 1E). A recent study has demonstrated that the IL-17RB pathway controls IL-9 expression in the context of allergic airway inflammation [31]. Furthermore, Tax has been shown to induce IL-9 expression and IL-9 can regulate the proliferation of primary ATL cells [42]. In light of these findings, we hypothesized that IL-9 may represent a key downstream gene of IL-17RB that governs the proliferation of HTLV-1 transformed T cells. First, to determine if IL-17RB regulated a soluble factor that was important for the proliferation of HTLV-1-transformed T cells, C8166 and MT-2 cells were transduced with lentiviruses expressing control or IL-17RB shRNAs and the media was then replaced with conditioned media from the corresponding cells. As expected, suppression of IL-17RB significantly reduced the proliferation of both C8166 and MT-2 cells (Figure 6A). However, the conditioned media rescued the proliferative defects associated with loss of IL-17RB (Figure 6A), suggesting that a soluble factor(s) is sufficient to restore the growth of these cells.

As described above, IL-9 represented an attractive candidate as a pro-proliferative soluble factor in the conditioned media from HTLV-1 transformed T cells. To determine if IL-9 was necessary to restore the proliferation of IL-17RB knockdown cells, we collected conditioned media from cells transduced with control or IL-9 shRNAs for the culture of C8166 cells expressing control or IL-17RB shRNA. Our results revealed that the conditioned media from cells with suppressed IL-9 expression was unable to restore the proliferation of C8166 cells expressing IL-17RB shRNA (Figure 6B). As expected, conditioned media from cells with control shRNA effectively restored C8166 cell growth (Figure 6B). IL-17RB and IL-9 knockdown were confirmed by qRT-PCR (Figure 6B). Next, to determine if IL-9 was sufficient to rescue the growth defect associated with suppressed IL-17RB expression we provided recombinant IL-9 to the media of HTLV-1 transformed cell lines expressing IL-17RB shRNA. The results indicated that provision of IL-9 was sufficient to restore cell proliferation of both C8166 and MT-2 cells (Figure 6C). Therefore, IL-9 is a key cytokine downstream of IL-17RB that governs the proliferation of HTLV-1-transformed T cells.

### TRAF6 is required for NF-κB activation in HTLV-1 transformed T cells

**Figure 7:**
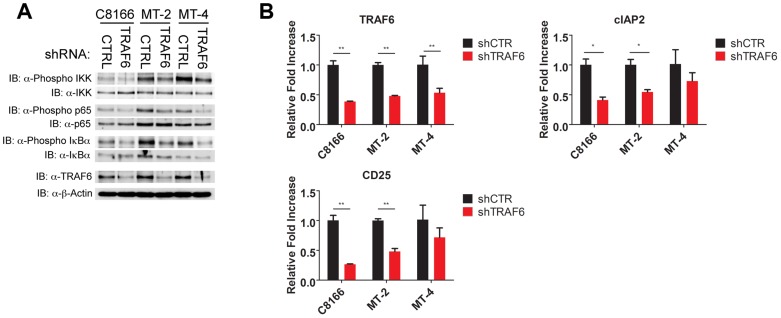
TRAF6 is required for NF-κB activation in HTLV-1 transformed T cells. (**A**) Western blots were performed with the indicated antibodies using whole cell lysates from C8166, MT-2 and MT-4 cells transduced with control or TRAF6 shRNA. (**B**) qRT-PCR of TRAF6, CD25 and cIAP2 mRNAs in C8166, MT-2 and MT-4 cells transduced with lentiviruses expressing control or TRAF6 shRNA.

IL-17RB can form a heterodimeric receptor complex together with IL-17RA [26], and upon binding to IL-25, the active receptor recruits the Act1 adaptor protein [28]. In addition, the ubiquitin ligase TRAF6 can be directly recruited to IL-17RB via a TRAF6 binding motif and plays a critical role in IL-17RB-mediated NF-κB activation and gene expression [30]. Given the vital role of IL-17RB in NF-κB signaling and survival of HTLV-1 transformed T cells, we sought to determine the requirements of the upstream signaling molecules that constitute this pathway. To this end, knockdown experiments were conducted in HTLV-1 transformed cell lines using shRNAs specific for TRAF6, IL-17RA and Act1. Interestingly, knockdown of TRAF6, but not IL-17RA or Act1, attenuated NF-κB activation as determined by western blotting for phosphorylated forms of IKK, p65 and IκBα (Figures 7A, S2C and S3C). Knockdown of TRAF6 also diminished the expression of NF-κB target genes CD25 and cIAP2 as shown by qRT-PCR in HTLV-1 transformed T-cell lines (Figure 7B). Knockdown of IL-17RA, but not Act1, modestly reduced the expression of CD25 and cIAP2 (Figures S2B and S3B). However, the proliferation of HTLV-1 transformed cell lines was strongly dependent on the expression of both IL-17RA and Act1 (Figures S2A and S3A). These results suggest that IL-17RA and Act1 regulate the proliferation of HTLV-1 transformed cells in an NF-κB independent manner. Since IL-17RA regulates chemokine mRNA stability independently of NF-κB [49], this mode of regulation may explain how IL-17RA and Act1 contribute to the proliferation of HTLV-1 transformed T cells.

### IL-17RB is essential for NF-κB signaling and survival in a subset of ATL cell lines lacking Tax expression

**Figure 8:**
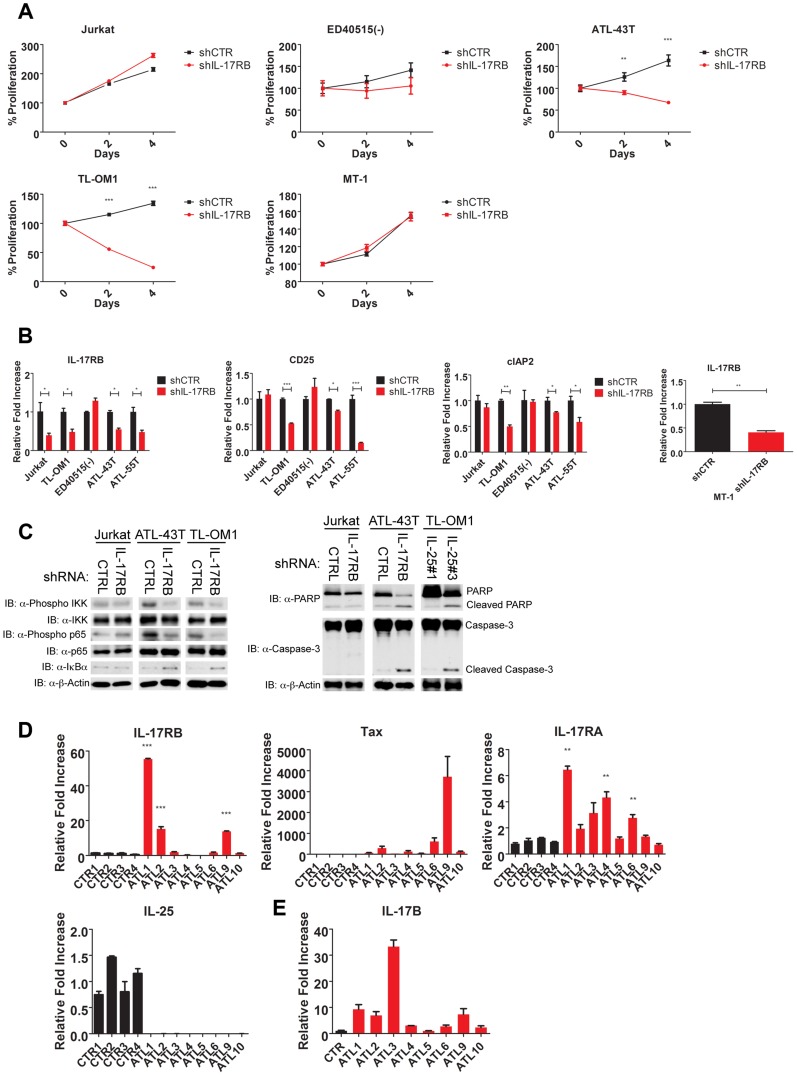
IL-17RB is essential for NF-κB activation and survival in a subset of Tax-negative ATL cell lines. (**A**) Proliferation/viability assay of the indicated cell lines transduced with lentiviruses expressing control or IL-17RB shRNA using CellTiter-Glo. (**B**) qRT-PCR of IL-17RB, CD25 and cIAP2 mRNAs in the indicated cell lines transduced with lentiviruses expressing control or IL-17RB shRNA. (**C**) Western blots were performed using whole cell lysates from the indicated cell lines transduced with control, IL-17RB or IL-25 shRNAs. (**D**) qRT-PCR of IL-17RB, Tax, IL-25 and IL-17RA mRNAs from control PBMCs (normal donors) or primary acute ATL leukemic cells. ATL mRNA expression was compared relative to the average of control mRNA expression. (**E**) qRT-PCR of IL-17B from purified CD4+ T cells (CTR) or primary acute ATL leukemic cells.

Thus far our experiments support a model of a Tax-induced IL-17RB-NF-κB feed-forward autocrine loop that is essential for the *in vitro* immortalization of primary T cells by HTLV-1 and the proliferation and survival of established HTLV-1 transformed T cell lines. However, the majority of ATL tumors (∼60%) have downregulated or lost Tax expression [19], and these malignant cells have acquired mechanisms to activate NF-κB persistently despite loss of Tax expression [50]. We next asked if IL-17RB played a role in NF-κB activation in ATL cells lacking Tax expression. Tax-negative ATL cell lines ATL-43T, ED40515(-), TL-OM1 and MT-1 were transduced with control or IL-17RB shRNA lentiviruses. Interestingly, proliferation and viability were significantly diminished in ATL-43T and TL-OM1, but not in ED40515(-), MT-1 and control Jurkat cells (Figure 8A). Knockdown of IL-17RB was efficient in all cell lines except ED40515(-), likely due to poor lentiviral transduction (Figure 8B). Thus, IL-17RB appears to be important for some, but not all Tax-negative ATL cell lines since MT-1 cells proliferated normally despite knockdown of IL-17RB (Figure 8A and B). The NF-κB target genes CD25 and cIAP2 were suppressed in TL-OM1 and ATL-43T cells, but not in the other ATL cell lines (Figure 8B). Phosphorylation of IKK and p65 was also inhibited by IL-17RB knockdown in ATL-43T and TL-OM1 cells (Figure 8C). The loss of NF-κB activation also triggered an apoptotic response as shown by PARP and caspase 3 cleavage (Figure 8C).

We next examined the expression of IL-17RB, IL-17RA, IL-25, IL-17B and Tax in eight primary acute ATL leukemic specimens. IL-17RB expression was significantly overexpressed in 3 out of 8 ATL samples compared to normal control PBMCs (Figure 8D). IL-17RA was modestly elevated in all the ATL samples compared to controls (Figure 8D). However, IL-25 expression was not detected in any of the ATL samples (Figure 8D). Tax mRNA was found in a subset of the samples but did not correlate with IL-17RB expression (Figure 8D), suggesting that ATL cells may regulate IL-17RB expression independently of Tax. Surprisingly, IL-17B was overexpressed in the majority of acute ATL samples (Figure 8E), thus raising the possibility that IL-17B may serve as a ligand for IL-17RB in acute ATL samples.

## Discussion

The IL-25-IL-17RB pathway has been linked to allergic airway inflammation and host defense against parasites. Our study has established a novel connection of this pathway to HTLV-1-induced leukemogenesis and also reveal that IL-17RB overexpression can be oncogenic in T cells. Tax promotes the aberrant expression of IL-17RB via NF-κB signaling to establish an IL-17RB-NF-κB feed-forward autocrine loop that drives persistent NF-κB activation in T cells, the natural host cell of HTLV-1. Therefore, Tax has hijacked the IL-17RB-NF-κB signaling axis to sustain high levels of NF-κB and coordinate the induction of a gene program consisting of inflammatory cytokines, chemokines and anti-apoptotic proteins that orchestrates pathogenic T-cell proliferation and survival. Together, these results provide a new framework for how Tax and HTLV-1 persistently activate NF-κB to promote the malignant transformation of T cells.

HTLV-1-induced leukemogenesis is a multi-step process that commences with the IL-2-dependent polyclonal expansion of HTLV-1 infected T cells. The Tax oncoprotein is thought to play critical roles in driving T-cell proliferation and survival in the early events of transformation by HTLV-1. However, at later stages Tax expression is largely dispensable, presumably due to genetic and epigenetic changes that may compensate for the loss of Tax. The HTLV-1-encoded HBZ protein may also exert oncogenic roles in ATL tumors in the absence of Tax expression [51]. Nevertheless, after loss of Tax expression, ATL tumors still exhibit constitutive canonical and noncanonical NF-κB signaling that sustains tumor cell proliferation and survival. However, the mechanisms of Tax-independent NF-κB activation in ATL tumors remain poorly understood. A recent study demonstrated that epigenetic downregulation of the microRNA miR-31 led to overexpression of NIK and activation of noncanonical NF-κB [52]. Our results reveal that IL-17RB drives canonical NF-κB signaling in a subset of ATL cell lines suggesting that the IL-17RB-NF-κB autocrine loop can be maintained in the absence of Tax, most likely by the acquisition of genetic and/or epigenetic changes. Comparative genomic hybridization (CGH) analysis has elucidated specific chromosomal imbalances associated with each of the clinical subtypes of ATL [53]. The highly aggressive acute ATL acquires more frequent chromosomal abnormalities, including characteristic gains at chromosomes 3p, 7q and 14q and losses at chromosomes 6q and 13q [54]. Interestingly, *IL-17RB* is encoded on chromosome 3p21.1, one of the most frequently amplified regions in acute ATL [54]. We found that IL-17RB is significantly overexpressed in leukemic cells from 3/8 ATL patients (38%), comparable to the 37% of aggressive ATL cases with 3p21 gains [54]. Therefore, IL-17RB overexpression in a subset of acute ATL tumors may potentially regulate the constitutive canonical NF-κB activation in the absence of Tax expression (Figure S4). Nevertheless, additional studies with more ATL patient tumor specimens are warranted to further explore the mechanisms underlying IL-17RB overexpression. It will also be interesting to determine if somatic mutations occur in *IL-17RB* that render the receptor constitutively active in the absence of ligand. Finally, since IL-17B but not IL-25, was expressed by acute ATL leukemic cells (Figure 8), future studies will need to examine if IL-17B plays a role in IL-17RB signaling, NF-κB activation and proliferation of primary ATL cells. IL-25 expression may potentially be suppressed by active mechanisms in ATL since it exerts pro-apoptotic roles in other tumor types [55].

IL-25 serves as the high affinity ligand for IL-17RB. IL-25 favors Th2 immune responses and orchestrates host defense against parasites by inducing the expression of IL-4, IL-5 and IL-13 [56]. IL-25 signals through a heterodimeric receptor containing IL-17RA/IL-17RB, which in turn recruits Act1 and TRAF6 upon IL-25 stimulation to induce NF-κB and MAPK activation that regulate genes important for Th2 immunity, allergic responses and expulsion of helminths. Our results indicate that HTLV-1 transformed cells are critically dependent on the IL-17RB pathway for proliferation, however only IL-17RB and TRAF6 are essential for NF-κB activation. IL-17RB contains a TRAF6 interaction motif in its intracellular domain that propagates downstream NF-κB activation [30]. Furthermore, a previous study has shown that TAK1, a kinase downstream of TRAF6 in the IL-17RB pathway, is also involved in Tax-mediated NF-κB activation [15]. Therefore, IL-17RB may signal through both TRAF6 and TAK1 to activate IKK in HTLV-1 transformed cells. We have recently identified a consensus TRAF6 interaction motif in the C-terminal region of Tax that mediates TRAF6 interaction and activation [57], thus suggesting that Tax may activate TRAF6 to further enhance the Tax-IL-17RB-NF-κB positive feedback loop in T cells. A previous study claimed that TRAF6 was dispensable for Tax-induced NF-κB activation [58], however they used a cell-free assay system using lysates from murine embryonic fibroblasts. Using intact T cells, we found that TRAF6 indeed plays a role in Tax-mediated NF-κB signaling. Our results also indicate that IL-17RB is dispensable for Tax to activate NF-κB in 293 cells, yet is critical for Tax-mediated NF-κB activation in T cells. Therefore, Tax activation of NF-κB appears to be distinct in T cells compared to other cell types and provides a strong rationale for Tax/NF-κB studies to be conducted in T cells.

Our data has provided new insight into the transcriptional regulation of IL-17RB. Little is known regarding how IL-17RB expression is regulated, although a previous study demonstrated that TGF-β and/or IL-4 can induce IL-17RB expression in mouse T cells [31]. We have provided multiple lines of evidence supporting a role for NF-κB in Tax-induced expression of IL-17RB. First, an IKKβ inhibitor greatly reduced the expression of IL-17RB in HTLV-1 transformed T cell lines (Figure 2A). Second, knockdown of IKKα or IKKβ with shRNAs diminished IL-17RB expression in C8166 cells (Figure 2B). Finally, the Tax M22 mutant, defective for NEMO binding and NF-κB activation, was impaired in the induction of IL-17RB (Figure 2F). Taken together, our data support a two-step model of Tax activation of NF-κB in T cells (Figure S4). First, Tax activation of canonical NF-κB commences through direct NEMO/IKK binding and IKK activation. The precise mechanisms remain poorly understood but may involve IKK oligomerization and inhibition of NEMO-associated phosphatase 2A [59], [60]. Next, Tax and IKK-induced IL-17RB overexpression (and engagement by IL-25) triggers downstream signaling to TRAF6 and further activates IKK to establish a positive feedback loop resulting in strong and sustained NF-κB signaling. It remains unclear whether NF-κB directly regulates the expression of IL-17RB, although we have identified a putative NF-κB site (GGGAATTTCC) ∼3380 base pairs upstream of the human *IL-17RB* transcriptional start site. Future studies will be necessary to identify important regulatory elements in the IL-17RB promoter.

IL-17RB forms heterodimers with IL-17RA, and although IL-17RA does not directly engage IL-25 it appears to be essential for IL-17RB signaling in untransformed cells [26]. Since IL-17RA and Act1 were largely dispensable for NF-κB activation in HTLV-1 transformed cells (Figures S2 and S3), it is plausible that IL-17RA and Act1 regulate the proliferation of these cells by stabilizing chemokine mRNAs [49]. Because IL-17RB is overexpressed to a much greater degree than IL-17RA in HTLV-1 transformed T cells, it is likely that IL-17RB homodimers constitute the most abundant IL-17R complex that signals to NF-κB in these cells. Further studies are needed to examine the stoichiometry of IL-17R complexes and downstream signaling requirements in HTLV-1 transformed cells.

Although IL-25/IL-17RB signaling has been previously linked to the induction of IL-9 and Th2 cytokines [24], our study has identified additional genes regulated by this pathway that contribute to oncogenesis. We found that knockdown of IL-17RB in HTLV-1 transformed cell lines diminished the expression of cytokines (IL-9), cytokine receptors (CD25), anti-apoptotic genes (cIAP2) and transcription factors (IRF4). Elevated expression of IRF4 in ATL tumors was shown to correlate with resistance to antiviral therapy with zidovudine (AZT) and interferon alpha [61]. Furthermore, cIAP2 was identified as a Tax regulated anti-apoptotic gene that was required for the survival of HTLV-1 transformed T cells [62]. IL-9 was also demonstrated to function as a key proliferative factor for ATL cells [42]. Consistently, we found that IL-9 was both necessary and sufficient to restore the cell proliferation of HTLV-1 transformed T cells with IL-17RB knockdown (Figure 6B and C). These data support the notion that IL-9 is an important downstream target gene of IL-17RB that drives the proliferation of HTLV-1 transformed cells. Additional studies will be necessary to identify the full spectrum of genes regulated by IL-17RB in HTLV-1 transformed T cells that support oncogenic proliferation.

Therapeutic blocking antibodies, such as those targeting HER2 and EGFR, have emerged as an important new treatment option in the clinic for carcinomas of the breast, lung and colon [63], [64]. IL-17RB is overexpressed in a subset of breast tumors and is associated with poor prognosis [34]. Treatment with blocking IL-17RB therapeutic antibodies attenuated the tumorigenicity of breast cancer cells [34]. Given that IL-17RB overexpression can promote oncogenic NF-κB signaling in Tax-negative ATL tumors, this receptor may represent an attractive therapeutic target for ATL. IL-17RB may potentially serve as a biomarker to stratify ATL patients that could benefit from IL-17RB inhibition. Preclinical studies with IL-17RB (or potentially IL-17B) monoclonal blocking antibodies in both *in vitro* and *in vivo* ATL models will be required to establish the feasibility of this potential targeted therapy.

## Materials and Methods

### Ethics statement

Blood from healthy donors was purchased from Biological Specialty Corporation (Colmar, PA). PBMCs were collected from acute ATL patients (n = 8). This study was conducted according to the principles expressed in the Declaration of Helsinki. The study was approved by the Institutional Review Board of Kyoto University (G204). All patients provided written informed consent for the collection of samples and subsequent analysis.

### Plasmids, cell lines, recombinant proteins and inhibitors

Human embryonic kidney cells (HEK 293T) and Jurkat T cells were purchased from ATCC. The HTLV-1-transformed cell lines MT-2, HUT-102, C8166 and MT-4 were described previously [65], [66]. ED40515(-), MT-1, and TL-OM1 cells are clones of leukemic cells derived from ATL patients, kindly provided by Dr. Michiyuki Maeda (Kyoto University). ATL43T is a Tax-negative ATL cell line that was previously described [67]. Jurkat Tax Tet-On cells were kindly provided by Dr. Warner Greene [45]. 293T cells were cultured in Dulbecco's Modified Eagle's medium (DMEM); Jurkat, MT-2, C8166, MT-4, ATL-43T, HUT-102, ED40515(-), MT-1 and TL-OM1 cells were cultured in RPMI medium. Media was supplemented with fetal bovine serum (FBS; 10%) and penicillin-streptomycin (1×). MISSION shRNAs targeting human IL-17RB, IL-17RA, IL-25, IL-9, Act1, CD40, OX40 and control scrambled shRNA were purchased from Sigma. TRAF6, Tax, IKKα and IKKβ shRNAs were cloned into pYNC352/puro. Target sequences for these shRNAs are listed in Table S3. Tax WT, M22 and M47 were cloned in the pDUET lentiviral vector. Expression vectors encoding κB Luciferase (Luc), pU3R-Luc, pRL-TK (thymidine kinase) have all been described previously [68]. Recombinant human IL-9 was purchased from R&D Systems. The IKKβ inhibitor SC-514 was from EMD Millipore.

### Antibodies

The following antibodies were used in this study: anti-hIL-17RB (FAB1207P; R&D Systems), anti-hCD4 (555346; BD Pharmingen), anti-hCD3 (552851; BD Pharmingen), anti-hCD8 (555366; BD Pharmingen), anti-hCD25 (560989; BD Pharmingen), anti-β-actin (AC15; Abcam), anti-IκBα (SC-371; Santa Cruz Biotechnology), anti-phospho-IκBα (14D4; Cell Signaling), anti-p65 (8242S; Cell Signaling), anti-phospho-p65 (3031S; Cell Signaling), anti-IKKβ (2678; Cell Signaling), anti-phospho-IKKα/β (2697S; Cell Signaling), anti-IL-17RB (SC-52925; Santa Cruz Biotechnology), anti-TRAF6 (SC-7221; Santa Cruz Biotechnology), anti-PARP (9542S; Cell Signaling) and anti-caspase-3 (SC-7148; Santa Cruz Biotechnology).

### Isolation of PBMCs

Human PBMCs from healthy donors were prepared from lymphocyte enriched human blood with a Ficoll-Hypaque gradient (Pharmacia Biotech). Samples were tested and found to be negative for hepatitis B virus (HBV), hepatitis C virus (HCV) and human immunodeficiency virus 1 (HIV-1). The cells were stimulated for 36 h with phytohemagglutinin (PHA, 2 µg/ml) and then cultured in RPMI medium supplemented with 20% FBS, 2 mM L-glutamine, penicillin-streptomycin, and 25 units/ml of human recombinant IL-2 (Biological Resources Branch, NCI). Under these conditions, PBMCs continuously grew for up to 4 weeks in the presence of exogenous IL-2. CD4+ T cells were isolated from PBMCs by negative selection using MACS MS Columns (Miltenyi Biotec). The purity of the cells was confirmed by flow cytometry and was>95%.

### *In vitro* transformation of T cells with HTLV-I

*In vitro* transformation of T cells with HTLV-I was performed as previously described [39]. Briefly, PHA-stimulated PBMCs were co-cultured with lethally γ-irradiated (50 Grays (Gy)) HTLV-1 donor cells (MT-2) in IL-2-containing RPMI medium. As expected, the virus-infected T cells became immortalized after about 6 weeks of co-cultivation. These cells proliferated vigorously when exogenous IL-2 was provided, a characteristic of T cells at an early stage of HTLV-1 infection. Under identical culture conditions, the uninfected control T cells or PBMCs typically ceased growth within 4 weeks, and the γ-irradiated MT-2 cells did not proliferate. The HTLV-1-immortalized T cells were maintained in RPMI medium supplemented with IL-2 and used as a bulk population. For shRNA knockdown studies, purified PHA-stimulated PBMCs were first infected with lentiviral particles expressing shRNAs to knockdown the indicated genes and subsequently co-cultured with lethally γ-irradiated (50 Gy) HTLV-1 donor cells (MT-2) in IL-2-containing RPMI medium. Puromycin was added after 3 weeks of co-culture to select for shRNA expressing cells.

### RNA-sequencing

Total RNA was prepared from parental primary T cells, HTLV-1-infected cells after 1 week of co-culture, HTLV-1 immortalized T cell clones after 12 weeks of co-culture or MT-2 cells. Dead cells were removed from co-cultured cells after magnetic labeling and separation using the Dead Cell Removal Kit (Miltenyi Biotec). RNA was isolated with RNeasy columns (Qiagen). RNA-Seq and bioinformatics were conducted by the Johns Hopkins Sidney Kimmel Cancer Center next-generation sequencing core. Sequencing analysis was performed by aligning the paired end reads to hg19 using Bioscope. The differential expression analysis was performed using the DEseq R package and the GO enrichment was done with the topGO R package.

### Transfections, lentiviral infections and luciferase assays

Jurkat cells were transfected with TransIT-Jurkat (Mirus) according to the manufacturer's instructions. For lentivirus production, HEK293T cells were transfected with a lentiviral vector and gag/pol-encoding plasmids using GenJet (SignaGen) according to the manufacturer's instructions. Virus was harvested after 48 h by centrifugation at 49,000× *g*. Cells were transduced with lentivirus by the spinoculation protocol, cultured for 48 h and then selected with puromycin. For luciferase assays, cells were lysed 24 h after transfection using passive lysis buffer (Promega). Luciferase activity was measured with the dual-luciferase assay system according to the manufacturer's instructions (Promega). Firefly luciferase values were normalized based on the *Renilla* luciferase internal control values. Luciferase values are presented as “fold induction” relative to the shControl (shCTR).

### Western blotting and immunoprecipitations

Western blotting was performed essentially as described previously [69]. Whole cell lysates were resolved by SDS-PAGE, transferred to nitrocellulose membranes, blocked in 5% milk or bovine serum albumin (BSA) (for phospho-specific antibodies), incubated with the indicated primary and secondary antibodies, and detected using Western Lightning enhanced chemiluminescence reagent (Perkin Elmer).

### Quantitative real time-PCR (qRT-PCR)

Quantitative real-time PCR (qRT-PCR) was performed as described previously [68]. Total RNA was isolated from cells using the RNeasy mini kit (Qiagen). RNA was converted to cDNA using the First Strand cDNA synthesis kit for RT-PCR (avian myeloblastosis virus [AMV]; Roche). Real-time PCR was performed using SYBR Green qPCR (Sigma). Gene expression was normalized to the internal control 18S rRNA. PCR primers are listed in Table S4.

### Cell viability and proliferation assays

Cell viability and proliferation assay was determined using the CellTiter-Glo Luminescent Cell Viability Assay (Promega). Cells were cultured in 96-well plates and the ATP content was quantified as an indicator of metabolically active cells.

### EMSA

Small-scale nuclear extracts were prepared from cells as described previously [47]. The following sequence was used to generate double-stranded oligonucleotides for electrophoretic mobility shift assays (EMSA): IL-2Rα NF-κB site: 5′-CAACGGCAGGGGAATCTCCCTCTCCTT. Nonradioactive EMSA was performed using LightShift Chemiluminescent EMSA Kit (Thermo Scientific) according to the manufacturer's instructions.

### Statistical analysis

Two-tailed unpaired T test was performed with Prism software. Error bars represent the standard deviation of triplicate samples. The level of significance was defined as: ***P<0.001, **P<0.01, *P<0.05.

## Supporting Information

Figure S1 **RNA-Seq analysis of IL-17RB in HTLV-1 infected and immortalized T cells.** Read coverage and mapping of *IL-17RB* on human genome *Hg19* chromosome 3 using the Integrative Genomics Viewer (Broad Institute). Upper panel represents parental primary T cells (W0), middle panel represents T cells co-cultured with irradiated MT-2 cells for 1 week (W1) and lower panel represents T cells immortalized by HTLV-1 after co-culture for 12 weeks (W12).
(PDF)

Figure S2 **IL-17RA regulates the proliferation of HTLV-1 transformed cell lines.** (**A**) Proliferation/viability assay of C8166, Jurkat, TL-OM1 and MT-2 cells transduced with lentiviruses expressing control or IL-17RA shRNA using CellTiter-Glo. (**B**) qRT-PCR of indicated mRNAs in C8166 cells transduced with lentiviruses expressing control or IL-17RA shRNA. (**C**). Western blots were performed with the indicated antibodies using whole cell lysates from MT-2 and MT-4 cells transduced with control or IL-17RA shRNAs. Error bars represent the standard deviation of triplicate samples. (***P<0.001, **P<0.01, *P<0.05).
(PDF)

Figure S3 **Act1 regulates the proliferation of HTLV-1 transformed cell lines.** (**A**) Proliferation/viability assay of C8166 cells transduced with lentiviruses expressing control or Act1 shRNAs using CellTiter-Glo. (**B**) qRT-PCR of CD25 and cIAP2 mRNAs in C8166 cells transduced with lentiviruses expressing control or Act1 shRNAs. (**C**). Western blots were performed with the indicated antibodies using whole cell lysates from MT-2 and MT-4 cells transduced with control or Act1 shRNAs. Error bars represent the standard deviation of triplicate samples. (*P<0.05).
(PDF)

Figure S4 **Model depicting the role of IL-17RB in HTLV-1-induced leukemogenesis.** 1) Tax interacts with IKK and upregulates the expression of IL-17RB. 2) Overexpression of IL-17RB synergizes with Tax to promote a feed-forward NF-κB activation loop. IL-17RA and Act1 do not appear to contribute to NF-κB activation but rather promote cell proliferation, possibly via enhanced chemokine and cytokine mRNA stability. 3) Loss of Tax in malignant ATL cells is associated with genomic instability and chromosome 3p gains in a subset of ATL patients resulting in the potential amplification of IL-17RB and the constitutive activation of NF-κB in the absence of Tax. IL-17B may serve as a ligand for IL-17RB in ATL since IL-25 is not expressed.
(PDF)

Table S1 **RNA-Seq results of primary T cells infected with HTLV-1.** A list of all transcripts that were upregulated or downregulated by more than 5 log2 fold change (*P*≤0.001) in primary T cells co-cultured with lethally irradiated MT-2 cells for 1 week.
(XLSM)

Table S2 **RNA-Seq results of primary T cells immortalized with HTLV-1.** A list of all transcripts that were upregulated or downregulated by more than 5 log2 fold change (*P*≤0.001) in primary T cells immortalized with HTLV-1 (primary T cells were co-cultured with lethally irradiated MT-2 cells for 12 weeks).
(XLSM)

Table S3 **Oligonucleotide sequences for shRNAs.**
(PDF)

Table S4 **Primer sequences for qRT-PCR.**
(PDF)
