# Supplementary figures and images for: A Critical Role for IL-17RB Signaling in HTLV-1 Tax-Induced NF-κB Activation and T-Cell Transformation
Source: PLoS Pathog. 2014 Oct 23;10(10):e1004418. doi: 10.1371/journal.ppat.1004418 (PMC4207800; doi:10.1371/journal.ppat.1004418)

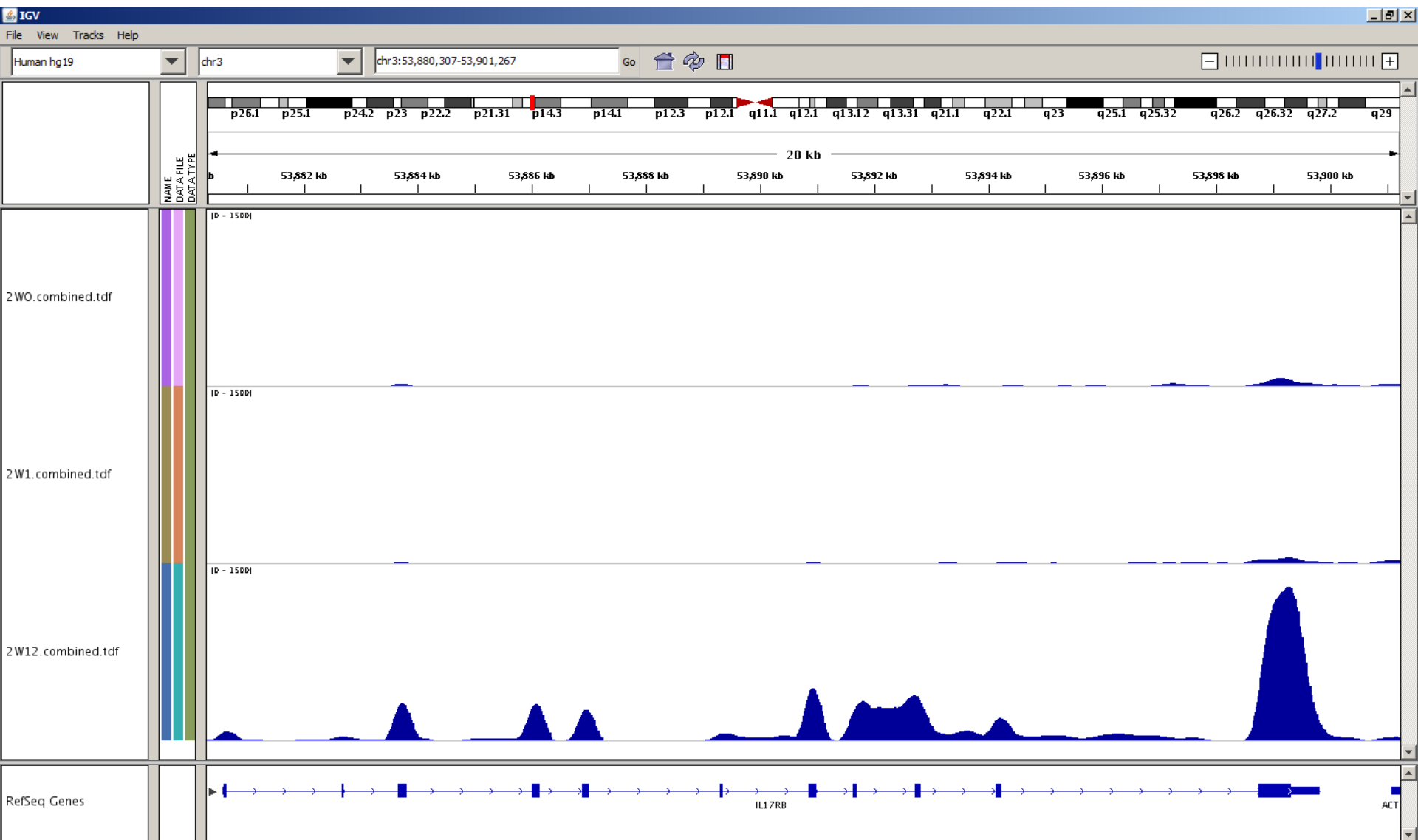

Supplement: Figure S1 — RNA-Seq analysis of IL-17RB in HTLV-1 infected and immortalized T cells. Read coverage and mapping of IL-17RB on human genome Hg19 chromosome 3 using the Integrative Genomics Viewer (Broad Institute). Upper panel represents parental primary T cells (W0), middle panel represents T cells co-cultured with irradiated MT-2 cells for 1 week (W1) and lower panel represents T cells immortalized by HTLV-1 after co-culture for 12 weeks (W12). (PDF) [file ppat.1004418.s001.pdf]

A

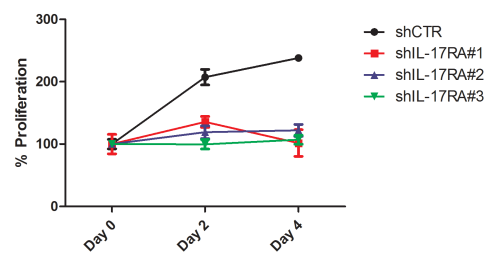

Jurkat

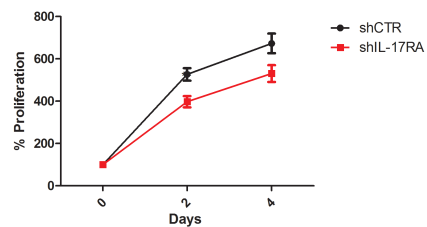

TL-OM1

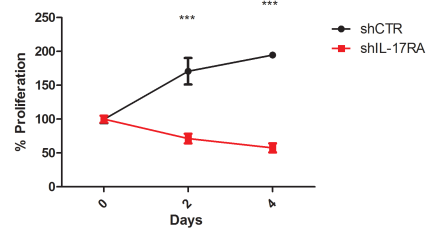

MT-2

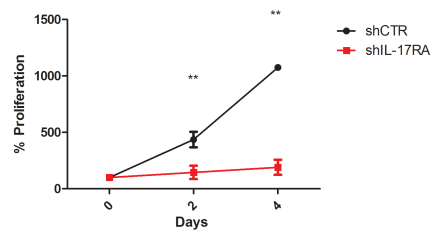

B

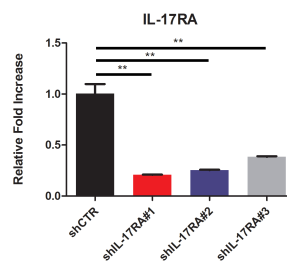

CD25

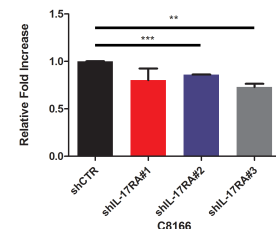

cIAP2

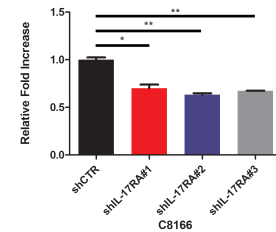

C

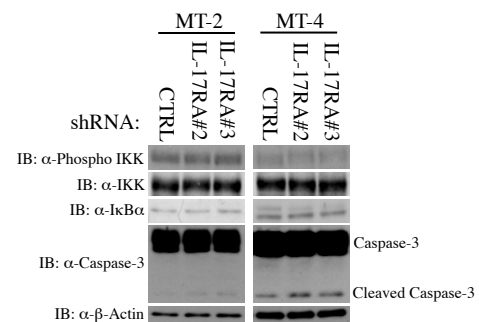

Supplement: Figure S2 — IL-17RA regulates the proliferation of HTLV-1 transformed cell lines. (A) Proliferation/viability assay of C8166, Jurkat, TL-OM1 and MT-2 cells transduced with lentiviruses expressing control or IL-17RA shRNA using CellTiter-Glo. (B) qRT-PCR of indicated mRNAs in C8166 cells transduced with lentiviruses expressing control or IL-17RA shRNA. (C). Western blots were performed with the indicated antibodies using whole cell lysates from MT-2 and MT-4 cells transduced with control or IL-17RA shRNAs. Error bars represent the standard deviation of triplicate samples. (***P<0.001, **P<0.01, *P<0.05). (PDF) [file ppat.1004418.s002.pdf]

A

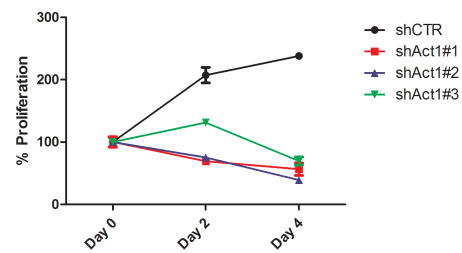

B

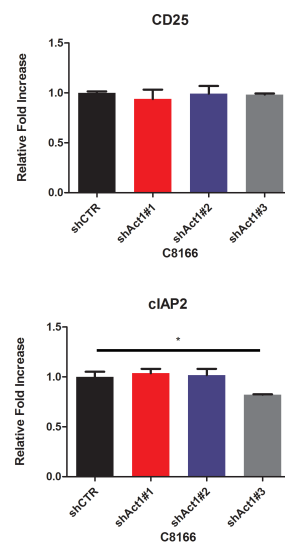

C

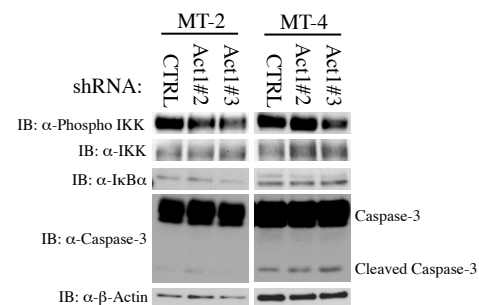

Supplement: Figure S3 — Act1 regulates the proliferation of HTLV-1 transformed cell lines. (A) Proliferation/viability assay of C8166 cells transduced with lentiviruses expressing control or Act1 shRNAs using CellTiter-Glo. (B) qRT-PCR of CD25 and cIAP2 mRNAs in C8166 cells transduced with lentiviruses expressing control or Act1 shRNAs. (C). Western blots were performed with the indicated antibodies using whole cell lysates from MT-2 and MT-4 cells transduced with control or Act1 shRNAs. Error bars represent the standard deviation of triplicate samples. (*P<0.05). (PDF) [file ppat.1004418.s003.pdf]
