# Supplementary material for: A Critical Role for IL-17RB Signaling in HTLV-1 Tax-Induced NF-κB Activation and T-Cell Transformation
Source: PLoS Pathog. 2014 Oct 23;10(10):e1004418. doi: 10.1371/journal.ppat.1004418 (PMC4207800; doi:10.1371/journal.ppat.1004418)
Supplement: Figure S4 — Model depicting the role of IL-17RB in HTLV-1-induced leukemogenesis. 1) Tax interacts with IKK and upregulates the expression of IL-17RB. 2) Overexpression of IL-17RB synergizes with Tax to promote a feed-forward NF-κB activation loop. IL-17RA and Act1 do not appear to contribute to NF-κB activation but rather promote cell proliferation, possibly via enhanced chemokine and cytokine mRNA stability. 3) Loss of Tax in malignant ATL cells is associated with genomic instability and chromosome 3p gains in a subset of ATL patients resulting in the potential amplification of IL-17RB and the constitutive activation of NF-κB in the absence of Tax. IL-17B may serve as a ligand for IL-17RB in ATL since IL-25 is not expressed. (PDF) [file ppat.1004418.s004.pdf]

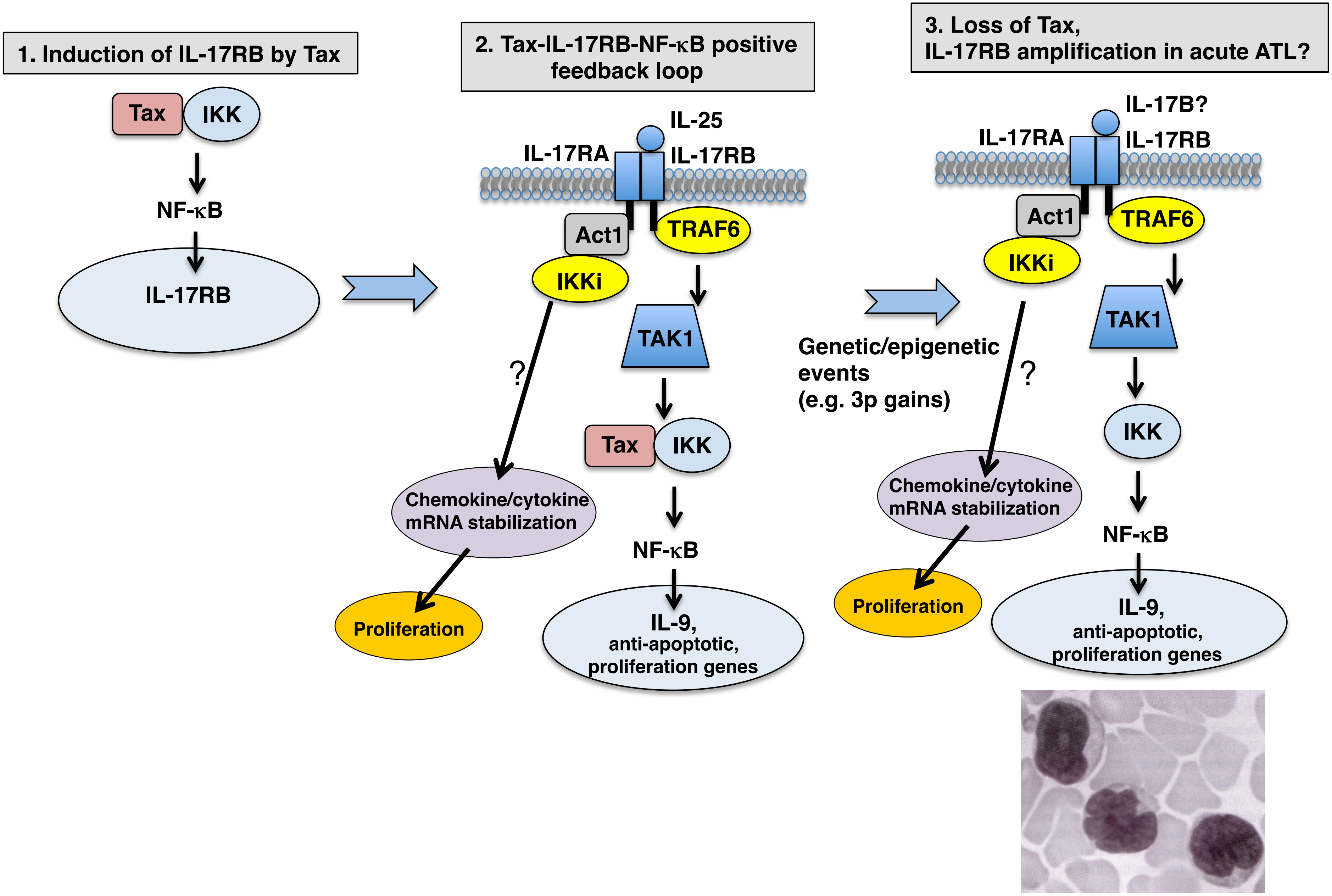
1. Induction of IL-17RB by Tax
Tax
IKK
NF-κB
IL-17RB
2. Tax-IL-17RB-NF-κB positive feedback loop
IL-25
IL-17RA
IL-17RB
Act1
TRAF6
IKKi
?
TAK1
Tax
IKK
Chemokine/cytokine mRNA stabilization
Proliferation
NF-κB
IL-9, anti-apoptotic, proliferation genes
3. Loss of Tax, IL-17RB amplification in acute ATL?
IL-17B?
IL-17RA
IL-17RB
Act1
TRAF6
IKKi
TAK1
Genetic/epigenetic events (e.g. 3p gains)
?
IKK
Chemokine/cytokine mRNA stabilization
Proliferation
NF-κB
IL-9, anti-apoptotic, proliferation genes
