# Supplementary material for: A Critical Role for IL-17RB Signaling in HTLV-1 Tax-Induced NF-κB Activation and T-Cell Transformation
Source: PLoS Pathog. 2014 Oct 23;10(10):e1004418. doi: 10.1371/journal.ppat.1004418 (PMC4207800; doi:10.1371/journal.ppat.1004418)
Supplement: Table S3 — Oligonucleotide sequences for shRNAs. (PDF) [file ppat.1004418.s007.pdf]

Table S3. Oligonucleotide sequences for shRNA.

Name	Target sequences
TRAF6 sh	5’- CCTGGATTCTACACTGGCAAA -3’
Tax sh3	5’- GCCTACCACCCCTCATTTCTA -3’
Tax sh5	5’- GCTTAGAGCCTCCCAGTGAAA -3’
IKKα sh1	5’- TGAATGTATTGCTGGATATAG -3’
IKKα sh4	5’- TGGCCATTTAAGCACTATTAT -3’
IKKβ sh3	5’- ACAGCGAGCAAACCGAGTTTG -3’
IKKβ sh4	5’- CATGAATGCCTCTCGACTTAG -3’
