# Supplementary material for: A Critical Role for IL-17RB Signaling in HTLV-1 Tax-Induced NF-κB Activation and T-Cell Transformation
Source: PLoS Pathog. 2014 Oct 23;10(10):e1004418. doi: 10.1371/journal.ppat.1004418 (PMC4207800; doi:10.1371/journal.ppat.1004418)
Supplement: Table S4 — Primer sequences for qRT-PCR. (PDF) [file ppat.1004418.s008.pdf]

Table S4. **Primer sequences for qRT-PCR.**

Name	Forward	Reverse
IL-17RB	5’- AGGGACCTCCGAGTAGAACC -3’	5’- CTTGGTGGCCTTCAACAAGC -3’
cIAP2	5’- TTTCCGTGGCTCTTATTCAAACT -3’	5’- GCACAGTGGTAGGAACTTCTCAT -3’
ISG15	5’- GCTCCATGTCGGTGTCAGAG -3’	5’- CTCGAAGGTCAGCCAGAACAG -3’
IFI27	5’- CATCAGCAGTGACCAGTGTG -3’	5’- CAATGGAGCCCAGGATGAAC -3’
OAS1	5’- CTCTTGCCAGACACGTGTTTC -3’	5’- GAGCGAACTCAGTACGAAGC -3’
MX1	5’- CTGTGCAGCCAGTATGAGGAG -3’	5’- CAGGGTGATTAGCTCATGACTG -3’
18S rRNA	5’- GTAACCCGTTGAACCCCATT -3’	5’- CCATCCAATCGGTAGTAGCG -3’
CCL1	5’- CCTGAGGGCAATCCTGTGTT -3’	5’- GTGCCTCTGAACCCATCCAA -3’
CXCR7	5’- CTGTGGGTTGTCCTCACCA -3’	5’- GACACGGCGTACCATCTTCT -3’
DDIT4L	5’- CAACTGGCAGTTTGAGCAGC -3’	5’- GACAGACAGTTCTCCAGCAT -3’
IL-25	5’- GCCTGTGCCTCCCCTAGAG -3’	5’- TATCTCCAGGGGGAGATGGC -3’
IL-9	5’- TCTGACAACTGCACCAGACC -3’	5’- TTGCATGGCTGTTCACAGGA -3’
IL-13	5’- TGTTGACCACGGTCATTGCT -3’	5’- GCATCCTCTGGGTCTTCTCG -3’
IL-17RA	5’- CTGATGGGGACCCAAACCAC -3’	5’- CCACAGGGTGAAGCTCACAC -3’
IL-17B	5’- CTGTGTCTGGGCTGTGTGAA -3’	5’- CACAGCGATGGTCTCCATGA -3’
CD25	5’- ATGCAAGAGAGGTTTCCGCA -3’	5’- AGTGGCAGAGCTTGTGCATTG -3’
IRF4	5’- GGATTGTTCCTGAGGGAGCC -3’	5’- AGGGTAAGGCGTTGTCATGG -3’
CD40	5’- CAGCCAGGACAGAAACTGGTGAGT -3’	5’-CTTCTTCACAGGTGCAGATGGTGTC-3’
OX40	5′- AACCAGGCCTGCAAGCCCT -3′	5′- GTCCCTGTCCTCACAGATT -3′
TRAF6	5'- TTTGCTCTTATGGATTGTCCCC -3'	5'- CATTGATGCAGCACAGTTGTC -3'
Tax	5’- ATCCCGTGGAGACTCCTCAA -3’	5’- CCAAACACGTAGACTGGGTATCC -3’
